# Purpose in Life and Its Measurement in Older Adults with Cognitive Impairment Using Cognitive Interviewing

**DOI:** 10.1093/geroni/igaf122.170

**Published:** 2025-12-31

**Authors:** Elise Hu, Maria Caceres, Lijuan Yin, Woojin Song, Kelsey King, Naoko Muramatsu

**Affiliations:** University of Illinois Chicago, Chicago, Illinois, United States; University of Illinois Chicago, Chicago, Illinois, United States; University of Illinois Chicago, Chicago, Illinois, United States; University of Illinois Chicago, Chicago, Illinois, United States; Roosevelt University, Chicago, Illinois, United States; University of Illinois Chicago, Chicago, Illinois, United States

## Abstract

Purpose in life (PiL), defined as the subjective experience of living with meaning, critically facilitates well-being in older adults with cognitive impairment (CI). The seminal PiL scale (Ryff, 1989) is the most widely used today and spans three domains: having a life purpose, engaging in that purpose, and setting purposeful goals. However, older adults with CI may interpret the scale items differently than intended. Guided by cognitive interviewing, a qualitative tool to verbalize thought processes behind response selection, this study examined how older adults with CI interpreted Ryff’s 7-item PiL scale to (1) identify purposes among and (2) assess its suitability for this population. 25 participants (ages 51-90) were enrolled in a physical activity trial designed for inactive older adults with mild CI or mild dementia. During baseline interviews, research staff displayed a 6-point Likert scale (strongly disagree to strongly agree) and read aloud each item. Participants responded and were asked to discuss their answer choice. Thematic analyses revealed that most participants identified purposes related to family. One item, “I sometimes feel as if I’ve done all there is to do in life,” misled participants because while feeling pride in completing life goals, they sensed the item’s undesirable overtone. Negatively worded items (e.g., “I don’t think”) confused participants with poorer cognition. PiL theory and measurement should thus account for various life stages and cognition levels. Future research will apply these findings to refine PiL measurement and explore how PiL can drive behaviors for healthy living among older adults with CI.

